# The complete mitochondrial genome of the firefly, *Asymmetricata circumdata* (Motschulsky) (Coleoptera: Lampyridae)

**DOI:** 10.1080/23802359.2016.1199000

**Published:** 2016-08-21

**Authors:** Xin Luan, Xinhua Fu

**Affiliations:** a Hubei Insect Resources Utilization and Sustainable Pest Management Key Laboratory, College of Plant Science and Technology, Huazhong Agricultural University, Wuhan, Hubei, China;; b Firefly Conservation Research Centre, No.1, Shizishan Street, Hongshan District, Wuhan, Hubei, China

**Keywords:** *Asymmetricata circumdata* (Motschulsky), firefly, lampyridae, mitochondrial genome

## Abstract

We report the complete mitochondrial genome of firefly, *Asymmetricata circumdata* (Motschulsky).The circular genome of 15,967 bp has a base composition of A (42.44%), C (12.83%), G (8.79%) and T (36.16%). Similar to other Metazoa, our sequence contains 13 protein-coding genes. All 13 protein-coding genes were initiated by the ATN (ATT, ATA and ATG) codon. Eight protein-coding genes stopped with TAA or TAG codon and the other 5 genes have an incomplete termination codon, a single T. We sequenced the mitochondrial genome of fireflies to analyze phylogenetic relationships and deduce the evolution of their flashing signals.

## Introduction

Fireflies have always been regarded as mysterious because of their bioluminescence. By using morphological characters, the genus *Asymmetricata* was erected in the Luciolinae in 2009 (Ballantyne & Lambkin 2009). *Asymmetricata circumdata* (Motschulsky) is distributed from Myanmar and Thailand to Cambodia (Ballantyne & Lambkin 2009). It is also widely distributed in adjacent Chinese provinces of Hainan, Guangxi, Jiangxi and Guangdong (Fu 2014).

Mitochondrial genome sequences are essential to a deeper understanding of the evolution of Lampyridae and other luminescent beetles (Ermakov et al. 2006). Here, we elucidate the mtDNA genome of *A. circumdata*.

Specimens were collected from Guangxi Province, China (23°24″N, 108°22″E) and were stored in Natural History Museum, Huazhong Agricultural University, Wuhan, Hubei, China (its accession no. is AC2014071301). As a species of steady-state bioluminescence fireflies, its habits, flashing signals and some morphology have been studied in detail (Wattanachaiyingcharoen et al. 2012; Goswami et al. 2015). However, there is no genetic research information about *A. circumdata*.

Specific primers were designed based on these conserved regions sequences. The PCR reaction was carried out with LA Taq polymerase for 35 cycles at 94 °C for 30 s, and annealed at 50 °C for 30 s, followed by extension at 72 °C for 1 min per 1 kb. Sequences were assembled using the software DNAstar v7.1 (Madison, WI) and adjusted manually to generate the complete sequence of mitochondrial DNA.

The complete mitochondrial genome sequence of *A. circumdata* (GenBank KX229747) has 15,967 bp and has a base composition of A (42.44%), C (12.83%), G (8.79%), T (36.16%). Similar to other Metazoa, our sequence contains 13 protein-coding genes, 22 transfer RNA genes, 2 ribosomal RNA genes and a non-coding AT-rich region, which represents a typical insect mitochondrial genome (Wolstenholme 1992). The open frames of the 13 protein-coding genes were inferred from three other fireflies: *Aquatica leii*, *Luciola substriata* and *Pyrocoelia rufa* (Lee et al. 2004; Jiao et al. 2013; Mu et al. 2015). All 13 PGGs initiated with ATN (ATT, ATA and ATG) codon, while 8 PGGs stopped with TAA or TAG codon, and the other 5 PGGs have an incomplete termination codon, namely, a single T (Table 1). The AT-rich region is 1388 bp, which is shorter than that of the other fireflies with reported sequences.

**Table 1.** Genes encoded by *Asymmetricata circumdata* mitochondrial genome.

		Position			Base composition (%)				Intergenic length*	Start codon	Stop codon
Gene	Direction	From	To	Size (bp)	A	C	G	T			
*tRNA^lle^*	F	1	64	64	39. 06	9. 38	17. 19	34. 38	0		
*tRNA^Gln^*	R	62	130	69	43. 48	14. 49	2. 90	39. 13	−3		
*tRNA^Met^*	F	130	194	65	33. 35	16. 92	10. 77	38. 46	−1		
*ND2*	F	195	1211	1017	41. 99	12. 68	7. 37	37. 95	0	ATA	TAA
*tRNA^Trp^*	F	1213	1278	66	39. 39	18. 18	13. 64	28. 79	1		
*tRNA^Cys^*	R	1352	1414	64	39. 06	10. 94	7. 81	42. 19	73		
*tRNA^Tyr^*	R	1414	1475	62	37. 10	16. 13	9. 68	37. 10	−1		
*COX1*	F	1468	3007	1540	32. 53	16. 04	14. 42	37. 01	−8	ATT	T + tRNA
*tRNA^Lou^*	F	3008	3071	64	34. 38	12. 50	14. 06	39. 06	0		
*C0X2*	F	3072	3750	679	36. 97	15. 17	11. 05	36. 82	0	ATA	T + tRNA
*tRNA^Lys^*	F	3751	3821	71	35. 21	16. 90	14. 08	33. 80	0		
*tRNA^Asp^*	F	3821	3886	66	50. 00	3. 03	3. 03	43. 94	−1		
*ATP8*	F	3887	4042	156	41. 03	11. 54	4. 49	42. 95	0	ATT	TAA
*ATPB*	F	4036	4710	675	36. 30	13. 04	9. 93	40. 74	−7	ATG	TAA
*C0X3*	F	4710	5493	784	33. 80	13. 52	14. 16	38. 52	−1	ATG	T + tRNA
*tRNA^Gly^*	F	5494	5556	63	47. 62	6. 35	6. 35	39. 68	0		
*ND3*	F	5557	5910	354	34. 46	12. 71	9. 89	42. 94	0	ATT	TAG
*tRNA^Aja^*	F	5909	5972	64	46.88	7. 81	7. 81	37. 50	−2		
*tRNA^Arg^*	F	5972	6035	64	42. 19	12. 50	10. 94	34. 38	−1		
*tRNA^Asn^*	F	6036	6100	65	49.23	10. 77	10. 77	29. 23	0		
*tRNA^Ser^*	F	6101	6163	63	39. 68	11. 11	9. 52	39. 68	0		
*tRNA^Glu^*	F	6164	6227	64	46.88	7. 81	3. 13	42. 19	0		
*tRNA^Phe^*	R	6226	6286	61	36. 07	13. 11	4. 92	45. 90	−2		
ND5	R	6287	7997	1711	48. 45	11. 86	8. 12	31. 56	0	ATA	T + tRNA
*tRNA^His^*	R	7995	8057	63	46.03	12. 70	3. 17	38. 10	−3		
ND4	R	8058	9381	1324	50. 76	12. 92	7. 93	28. 40	0	ATG	T + tRNA
ND4L	R	9375	9665	291	50. 86	13. 40	6. 53	29. 21	−7	ATG	TAA
*tRNA^Thr^*	F	9667	9728	62	45. 16	6. 45	8. 06	40. 32	1		
*tRNA^Pro^*	R	9729	9792	64	39. 06	14. 06	6. 25	40. 63	0		
*ND6*	F	9797	10279	483	39. 75	12. 84	7. 66	39. 75	4	ATA	TAA
*cytB*	F	10279	11412	1134	34. 57	14. 99	11. 55	38. 89	−1	ATG	TAG
*tRNA^Ser^*	F	11411	11477	67	43. 28	7. 46	11. 94	37. 31	−2		
*ND1*	R	11494	12426	933	50. 16	14. 26	9. 75	25. 83	16	ATT	TAG
*tRNA^Lou^*	R	12446	12506	61	40. 98	18. 03	8. 20	32. 79	19		
*16s rRNA*	R	12507	13770	1264	46.52	12. 10	5. 85	35. 52	0		
*tRNA^Vol^*	R	13771	13839	69	42. 03	13. 04	7. 25	37. 68	0		
*12s rRNA*	R	13840	14579	740	44. 32	13. 51	6. 22	35. 95	0		
*AT-rich region*		14580	15967	1388	44. 81	7. 71	2. 74	44. 74			

**Figure 1.**
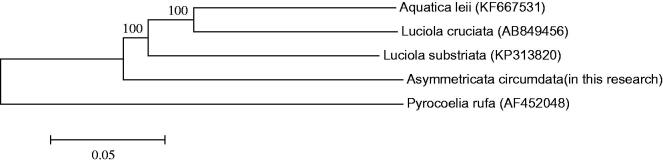
Molecular phylogeny of *Asymmetricata circumdata* and four other firefly species based on the complete mitochondrial genome. The complete mitochondrial genome was downloaded from GenBank and the phylogenic tree was constructed by Neighbour-Joining method with 1000 bootstrap replicates. MtDNA accession numbers used for tree construction are as follows: *Aquatica leii* (KF667531), *Luciola cruciata* (AB849456), *Luciola substriata* (KP313820), *Pyrocoelia rufa* (AF452048).

The phylogenetic tree among the five species based on mitochondrial genome sequences were aligned in MEGA 5 (Phoenix, AZ) (with 1000 bootstrap replicates) to construct a Neighbour-Joining tree (Figure 1).

The result shows *A*. *circumdata* is most closely related to *L. substriata*, which belongs to an entirely different genus in the Lampyridae.

In conclusion, the complete mitochondrial genome sequence of *A. circumdata* provides an important molecular framework for further phylogenetic analyses of fireflies. These data are essential for deeper understanding of the role of sexual and natural selection in the evolution of firefly flashing signals.
